# Nurse Practitioners' Use of Communication Techniques: Results of a Maryland Oral Health Literacy Survey

**DOI:** 10.1371/journal.pone.0146545

**Published:** 2016-01-14

**Authors:** Laura W. Koo, Alice M. Horowitz, Sarah D. Radice, Min Q. Wang, Dushanka V. Kleinman

**Affiliations:** 1 Department of Organizational Systems and Adult Health, School of Nursing, University of Maryland Baltimore, Baltimore, MD, United States of America; 2 Department of Behavioral and Community Health, School of Public Health, University of Maryland College Park, College Park, MD, United States of America; 3 Office of the Dean, School of Public Health, University of Maryland College Park, College Park, MD, United States of America; 4 Department of Epidemiology and Biostatistics, School of Public Health, University of Maryland College Park, College Park, MD, United States of America; UNC School of Dentistry, University of North Carolina-Chapel Hill, UNITED STATES

## Abstract

**Objectives:**

We examined nurse practitioners’ use and opinions of recommended communication techniques for the promotion of oral health as part of a Maryland state-wide oral health literacy assessment. Use of recommended health-literate and patient-centered communication techniques have demonstrated improved health outcomes.

**Methods:**

A 27-item self-report survey, containing 17 communication technique items, across 5 domains, was mailed to 1,410 licensed nurse practitioners (NPs) in Maryland in 2010. Use of communication techniques and opinions about their effectiveness were analyzed using descriptive statistics. General linear models explored provider and practice characteristics to predict differences in the total number and the mean number of communication techniques routinely used in a week.

**Results:**

More than 80% of NPs (N = 194) routinely used 3 of the 7 basic communication techniques: simple language, limiting teaching to 2–3 concepts, and speaking slowly. More than 75% of respondents believed that 6 of the 7 basic communication techniques are effective. Sociodemographic provider characteristics and practice characteristics were not significant predictors of the mean number or the total number of communication techniques routinely used by NPs in a week. Potential predictors for using more of the 7 basic communication techniques, demonstrating significance in one general linear model each, were: assessing the office for user-friendliness and ever taking a communication course in addition to nursing school.

**Conclusions:**

NPs in Maryland self-reported routinely using some recommended health-literate communication techniques, with belief in their effectiveness. Our findings suggest that NPs who had assessed the office for patient-friendliness or who had taken a communication course beyond their initial education may be predictors for using more of the 7 basic communication techniques. These self-reported findings should be validated with observational studies. Graduate and continuing education for NPs should increase emphasis on health-literate and patient-centered communication techniques to increase patient understanding of dental caries prevention. Non-dental healthcare providers, such as NPs, are uniquely positioned to contribute to preventing early childhood dental caries through health-literate and patient-centered communication.

## Introduction

This study provides an assessment of the communication techniques of nurse practitioners (NPs) in Maryland, as part of a statewide oral health literacy assessment of dentists, dental hygienists, physicians, nurse practitioners, and the public [1–7]. The assessment focused on prevention of dental caries in young children and included: surveying healthcare providers’ use of effective communication techniques; providers’ knowledge and opinions about preventing oral disease; environmental surveys of dental clinics; and surveys of the public’s understanding of how to prevent dental caries. Maryland adults with children younger than 6 years old in the household demonstrated low oral health literacy; they reported limited understanding of how to prevent early childhood caries [5]. Maryland physicians, dentists, and dental hygeinists report using some recommended health-literate communication techniques routinely, more so when they report taking a communication course outside of their original professional training [2,4,6].

Oral health literacy is a term which refers to a match in the skills of the public and the demands of the healthcare providers and systems to optimize oral health and to prevent dental caries [3,8]. Improving the nation’s oral health means reducing or eliminating the mismatch between the abilities of the public to obtain, understand, and act upon oral health information, and the expectations and characteristics of healthcare providers and healthcare systems who provide oral health information and services [3,9].

Understanding what the public and providers know and how they communicate is important during the years after the preventable 2007 death of a Maryland child from complications related to severe tooth decay[10]. Dental caries is a preventable chronic disease, and the most common childhood disease [11,12]. If it develops and progresses untreated, dental caries lead to pain, whole and partial tooth loss, systemic infection, impaired growth and development, nutritional deficiency, and in the worst of scenarios—death [10,12,13]. A nationwide dental crisis exists in the United States (U.S.): vulnerable populations experience lack of access to comprehensive dental care, lack of adequate dental insurance coverage, and low oral health literacy [14].

One of several proposed solutions to the nationwide dental crisis includes use of traditionally non-dental healthcare professionals such as nurses, pharmacists, and physicians, and social service providers to screen for oral health disease, provide education, and provide preventive oral health services [14]. These non-dental healthcare provider activities are imperative because there is a gap in timeframe between the recommended age of one year for establishing a dental home and when caregivers of children typically establish a dental home [15,16]. Nurse practitioners in the U.S. are advanced practice nurses who can diagnose, treat, and educate patients about common acute and chronic health conditions in a variety of inpatient and outpatient settings [17]. Healthy, decay-free teeth require appropriate use of topical fluorides (toothpaste, mouth rinses and professionally applied fluorides), consumption of systemic fluoride (fluoridated water or dietary fluoride drops or tablets), dental sealants; good oral hygiene habits; and limited consumption of refined carbohydrates [12]. Non-dental healthcare providers, such as NPs, can intervene early through counseling, screenings, and providing fluoride varnish [14]. They must use effective communication techniques and must promote a health-literate organizational environment to actively engage the patient and/or caregiver in preventive oral self-care, dental screenings, and clinical care. Health-literate and patient-centered communication strategies are highlighted to improve care delivery through U.S. healthcare reform initiatives such as the Patient Protection and Affordable Care Act (ACA) [18–21]. Leading U.S. healthcare agencies and Healthy People 2020 recommend health-literate and patient-centered care to improve patient safety and quality of care, as well as to support care at home [22–25]. Patient-centered communication, also referred to as the biopsychosocial communication style, includes: open-ended questions, information-seeking, information-giving, partnership-forming, confirming comprehension, positive talk, and seeking the patient’s perspective related to etiology and treatment [26,27]. A patient-centered provider bears the responsibilities of helping ensure patients have accurate, accessible and usable information, as well as the support needed to make decisions and participate in their own care [28]. Patient-centered communication is associated positively with patient satisfaction, increased adherence to treatment plans, and improved health [27,29–31]. A qualitative analysis of a convenience sample of NP-patient encounter transcripts revealed that approximately 30% of NPs used the patient-centered communication technique of “information-giving”, while 70% used a provider-centered communication method [26].

The American Medical Association and health literacy experts recommend using 17 communication techniques to improve patient-provider communication [32]. Key communication strategies in include speaking slowly, using simple “living room” language, limiting to 2 or 3 messages at a time, and confirming understanding with the teach-back method [32,33]. Simple “living room” language, or plain language, is clear, straightforward communication which avoids complicated vocabulary and sentence structure so that the audience can understand the message the first time that they read it or hear it [34]. This “health literacy universal precautions” approach to delivering healthcare that recommends that providers integrate clear, easy-to-understand communication into healthcare delivery much in the same way that healthcare providers use “universal precautions” when handling blood and bodily fluids [33].

The teach-back method checks patient understanding by asking how a patient would follow the instructions at home, while conveying that the provider is responsible for communicating the message clearly [33]. The teach-back method has demonstrated improved patient recall, comprehension in understanding immunizations and consent forms, and improved disease-specific outcomes in patients with diabetes and heart failure [24,35–38]. Checking patient understanding is so important that Healthy People 2020 contains a national public health objective “to increase the proportion of persons who report their health care provider always asked them to describe how they will follow the instructions” [25].

Health-literate organizations are institutions which make it easy for people to “navigate, understand, and use” health information and services in order to care for themselves [9]. Health-literate organizations strategically integrate health literacy concepts into measures of quality and quality improvement, as well as in assessing the facility’s environment for user-friendliness. Assessments for user-friendliness may include looking for use of clear symbols on signage, ease of facility navigation, and literacy levels of patient education materials and patient forms [3,39]. The purpose of this study was to determine the routine use of recommended health-literate and patient-centered communication techniques and the perceived effectiveness of those techniques by nurse practitioners in Maryland as part of a state-wide oral health literacy assessment [1–7].

## Materials and Methods

A 27 item survey containing 17 items on use of communication techniques was used in this descriptive study of nurse practitioners in Maryland. The University of Maryland College Park’s Institutional Review Board (ethics committee) approved the study. The questionnaire consisted of two parts. The first part addressed nurse practitioners knowledge, understanding and practices regarding prevention of dental caries which was adapted from previously used surveys [40,41]. The second part of the survey, the focus of this manuscript, addressed the reported use of recommended communication techniques and the perceived effectiveness of those techniques.

The 17 survey items on communication were adapted from a survey conducted by the American Dental Association [42], which was based on a communications techniques survey developed by the American Medical Association [32]. The 17 items are grouped into five domains. Two of the domains, Interpersonal Communication and Teach-Back Methods, consist of the seven basic communication techniques considered basic skills that all healthcare providers should routinely use. The other 10 items are also considered useful, especially for those with low literacy skills [33]. Participants were asked to indicate on a five point Likert-like scale (1 being “never” to 5 being “always”) their frequency of use of each technique during a typical work week. They also were asked to indicate their opinions about whether they believed the technique to be effective by responding: “yes,” “no,” or “don’t know”, a proxy for outcome expectancy, which influences performing a behavor, according to social cognitive theory [43].

Respondents were additionally asked if they had assessed their clinical practice for user-friendliness for patients. Another item asked if they were interested in taking a communication course in the future. Race/ethnicity was assessed with one item in which participants voluntarily selected among the following options: white, black, Hispanic, Asian/Pacific Islander, American Indian/Native Alaskan, or other/please specify. Race/ethnicity and gender were assessed to examine possible provider attributes potentially associated with regular use of communication techniques.

To ensure content validity, two pediatric dentists, two public health dentists, and one cariologist (specialist in dental caries) reviewed the draft questionnaire. A pilot test was conducted with dentists to assure the understandability of questions, and to gain agreement on the correct responses. Subsequently, it was pilot tested among six clinicians with a nursing background for understandability. No revisions were necessary for communication items; wording of some items about knowledge of dental caries prevention was edited minorly based on the pilot testing.

The list of all nurse practitioners licensed in Maryland was purchased from the Maryland Board of Nursing. In August and September 2010, the 27-item questionnaire was mailed to all 1,410 nurse practitioners who were identified with the licensing board as pediatric nurse practitioners, family practice nurse practitioners, or women’s health (obstetric/gynecologic) nurse practitioners. These NP specialties were chosen because they care for patients during developmental periods of young teeth: pregnancy, infancy, and early childhood. To facilitate easy return of the survey, it was designed to be postage pre-paid and pre-addressed.

Respondents were contacted three times by mail to boost response rate. Three sequential mailings were sent approximately three weeks apart. The first and second mailings contained the survey instrument with a cover letter by the then president of the Nurse Practitioners Association of Maryland (NPAM). The third mailing was a postcard sent to all non-respondents reminding them to complete the survey.

### Data analysis

The outcome variable analyzed was the “routine use of communication techniques” as defined by responses of “most of the time” or “always” as was done in similar studies of dentists and dental hygienists [4,6]. The data was analyzed using SPSS v.21. Statistical analyses included descriptive, cross tabulations and chi-square. General linear models were used as follows: analysis of variance with the dependent variable of “mean number of communication techniques routinely used per week” and ordinary least squares regression with the dependent variable of “number of communication techniques routinely used per week”. Provider characteristics and practice characteristics were the independent variables in each of the models. Models were run for the 17 communication techniques and for the 7 basic communication techniques. P-values were selected at the .05 level due to the exploratory nature of the study.

## Results

### Description of sample

Of the 1,410 surveys mailed to all pediatric, family, and women’s health nurse practitioners in Maryland, the response rate was 20.57% (n = 290), with effective response rate of 18.65% (n = 263) after excluding all un-usable responses. Almost all of the respondents were female (93.8%) and white (85.8%). The respondents practiced in a variety of settings, with the largest proportion in group practices (35%) (Table 1). Respondents represented a wide range of almost four decades of professional practice since graduation from their nursing programs. Seventy-seven percent (n = 171) of NPs saw children as part of their practice. Of those NPs who saw children, 57% reported Medicaid was the primary pediatric dental insurance; 39% reported private insurance was the primary dental insurance; and 4% reported out-of pocket was the primary dental payment. Sixty-five percent (n = 149) of all respondents reported ever taking a communication course in addition to their nursing education. Nurse practitioners who had taken a communications course beyond their nursing education were more likely to also have assessed their office to determine how user-friendly it is for patients (Chi-square = 26.87, p < .0001, N = 207).

**Table 1:** Characteristics of Nurse Practitioner Survey Respondents.

Characteristic	N^a^	Percentage^b^
***Year of graduation***		
**1961–1977**	58	25.89
**1978–1987**	58	25.89
**1988–1997**	50	22.32
**1998–2009**	58	25.89
***Race/Ethnicity***		
**White**	193	85.78
**Black**	20	8.89
**All other**	12	5.33
***Gender***		
**Female**	213	93.83
**Male**	14	6.17
***Ever taken a communication course***		
**Yes**	149	65.07
**No**	80	34.93
***Assessed office for user friendliness***		
**Yes**	114	55.07
**No**	93	44.93
***Inclusion of child patients in clinical practice***		
**Yes**	171	77.03
**No**	51	22.97
***Primary type of dental insurance of child patients***		
**Medicaid/SCHIP**	131	57.21
**Private insurance**	89	38.86
**Out of pocket**	9	3.93
***Practice setting***		
**Solo practice**	19	8.33
**Group practice**	80	35.09
**Public health**	39	17.11
**Private hospitals**	34	14.91
**All other**	56	24.6

^a^Total N = 263. May not add up to 263 due to missing values.

^b^May not add up to 100% due to rounding.

### Descriptive results for communication techniques routinely used

The 17 communication items are grouped into 5 domains: interpersonal communication, teach-back, patient-friendly materials and aids, assistance, and patient-friendly practice. The 7 basic communication techniques are comprised of the 7 items in the two domains of interpersonal communication and teach-back. The distribution of responses across the five-point Likert-like scale (never, rarely, occasionally, most of the time, always) varied greatly across items and domains, yet the modes of responses provide some generalization about trends (Table 2). Two items had the modes of the highest percentage of responses in the “always” use category of communication techniques: the use of simple language (50.5%) and the use of a translator or interpreter when needed (31.4%). The modes in the “most of the time” use category included 8 out of the 17 communication techniques; the modes for an additional 6 communication techniques were in “occasionally”. The modes for the two teach-back items were “most of the time” for asking patients to repeat back instructions (41.2%) and for asking patient to describe how they would follow instructions at home (38.1%). The only mode for highest percentage of responses in the “never” use category of communication techniques was for using video or DVD (53.4%).

**Table 2:** Percent Distribution of Communication Techniques Routinely Used by Nurse Practitioners.

Domain & Item	N	Always (%)	Most of the time (%)	Occasionally (%)	Rarely (%)	Never (%)	Mean Score (1–5)^b^
***Interpersonal communication***							
**Limit number of concepts presented at a time to 2–3**^a^	192	13.54	**67.71**	15.10	3.13	0.52	3.90
**Ask patients whether they would like a family member or friend to accompany them in the discussion**^a^	192	3.65	19.27	**40.63**	23.44	13.02	3.54
**Draw pictures or use printed illustrations**^a^	193	12.44	31.09	**39.90**	15.03	1.55	3.37
**Speak Slowly**^a^	194	20.62	**61.86**	16.49	0.52	0.52	4.02
**Use simple language**^a^	194	**50.52**	45.36	4.12	0	0	4.41
***Teach-back***							
**Ask patients to repeat back information or instructions**^a^	194	11.86	**41.24**	34.54	9.79	2.58	3.50
**Ask patients to tell you what they will do at home to follow instructions**^a^	194	16.49	**38.14**	30.93	11.86	2.58	3.54
***Patient-friendly materials and aids***							
**Use video or DVD**	193	0.52	3.11	9.33	33.68	**53.37**	2.65
**Hand out printed materials**	192	30.21	**43.75**	22.40	3.13	.52	4.00
**Use models or x-rays to explain**	194	4.64	17.53	**40.72**	22.68	14.43	2.75
***Assistance***							
**Underline key points on print materials**	193	21.24	**35.23**	24.35	15.03	4.15	3.54
**Follow-up with patients by telephone to check understand and adherence**	193	4.66	13.99	**38.86**	26.94	15.54	2.77
**Read instructions out loud**	193	23.32	**40.41**	22.28	8.81	5.18	3.67
**Ask other office staff to follow-up with patients for post-care instructions**	193	6.22	16.06	**34.72**	26.94	16.06	2.69
**Write or print out instructions**	194	31.96	**41.75**	20.62	4.12	1.55	3.98
***Patient-friendly practice***							
**Refer patients to the internet or other sources of information**	193	5.18	24.87	**50.26**	15.03	4.66	3.10
**Use a translator or interpreter when needed**	191	**31.41**	20.94	23.56	16.23	7.85	3.51

**Bold** = Mode.

^a^Seven basic communication techniques.

^**b**^Mean score (1 = Never, 5 = Always).

**Fig 1:**
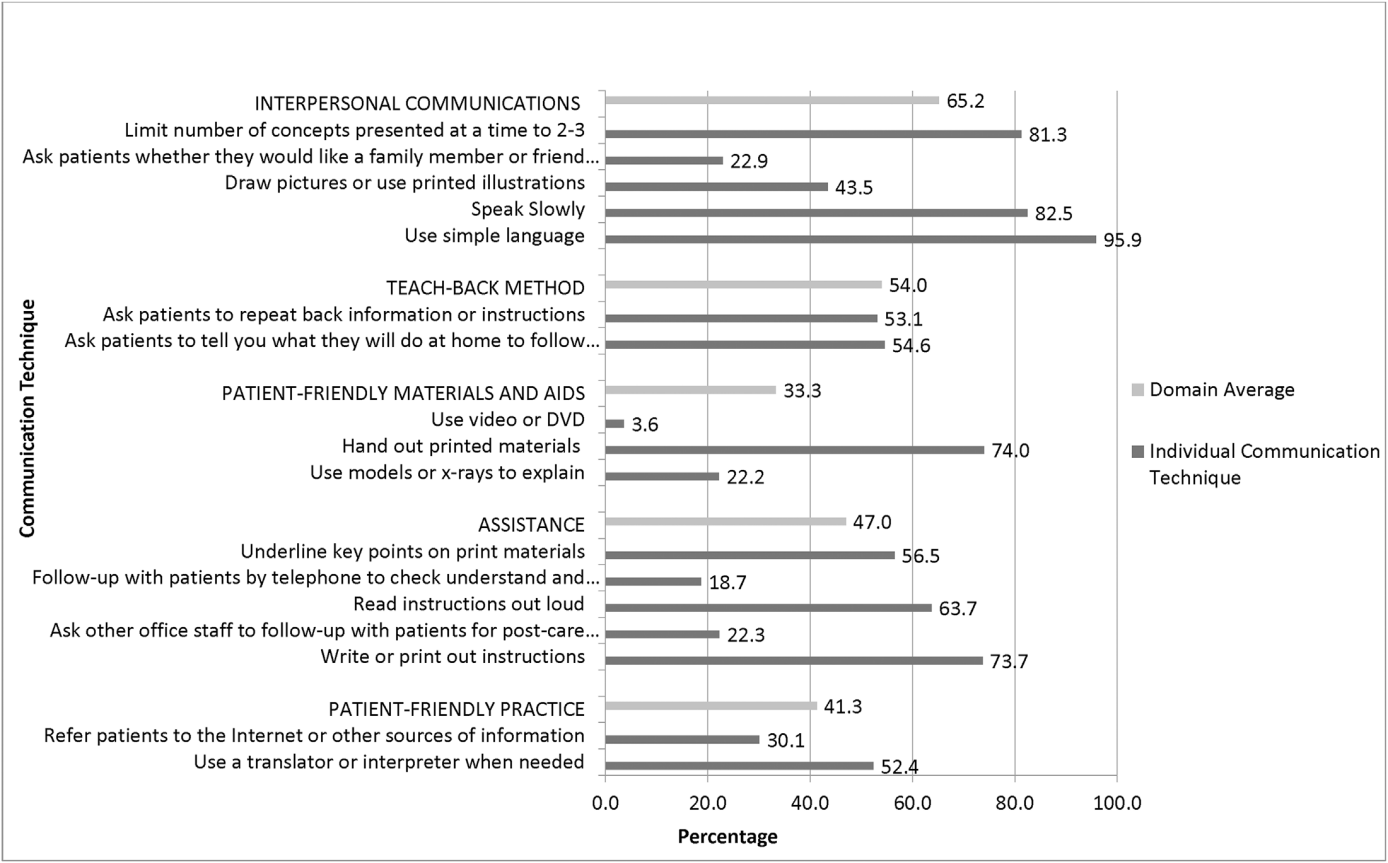
Percentage of Nurse Practitioners Reporting Routine Use of Each Communication Technique.

Routine use of communication techniques reported by NPs was defined as responses of “always” or “most of the time”. Of the 7 basic communication techniques, more than 80% of NPs reported routinely used the following 3 techniques: limiting to 2 or 3 concepts presented, speaking slowly, and using simple language. Nearly all (95.9%) reported routine use of simple language (Fig 1). An average of 54.0% of the NPs reported use of the teach-back method (with two items in that domain). For the additional items that comprise the 17 communication techniques, 74.0% of NPs reported routinely handing out printed materials, and 73.7% reported routinely writing or printing out instructions, with 56.5% routinely underlining key points on written materials and 66.7% routinely reading instructions aloud. In contrast, 22.92% of respondents report routinely asking patients whether they would like a family member or friend to accompany them for the discussion; 22.17% routinely used models or x-rays; 43.5% routinely drew pictures or used printed illustrations; and 3.6% routinely used video or DVD. Nineteen percent of NPs reported routinely followed up with patients by telephone to check understanding and adherence, and 22.3% routinely asked office staff to follow-up with patients for post-care instructions.

During a typical work week, NPs reported routinely using an average of 4 of the 7 basic communication techniques and 8 of the 17 communication techniques. Sixty-six percent of NPs reported routine use of 4 or more of the 7 basic communication techniques. Forty percent of NPs reported routine use of 10 or more of the 17 communication techniques.

### Nurse practitioners’ opinions about the effectiveness of recommended communication techniques

More than 75% of survey respondents believed that 6 of the 7 basic communication techniques are effective, provided the choices about effectiveness of “yes/effective”, “no/not effective”, and “don’t know” (Table 3). Sixty-two percent of the NPs expressed the opinion that they did not know whether the seventh technique, asking patients whether they would like a family member or friend to accompany them in the discussion, is effective. Of the additional techniques included in the 17 communication techniques, more than 79% of NPs believed that writing or printing out instructions and using a translator or interpreter when needed is effective. More than 62% expressed the opinion that they did not know the effectiveness of the following 4 techniques: using a video or DVD, handing out printed materials, underlining key points on print materials, and following-up with patients by telephone to check understanding and adherence. The most divided NP opinions were about two communication techniques: the effectiveness of asking other office staff to follow-up with patients for post-care instructions (yes/effective 54%; no/not effective 5%; don’t know 41%) and about referring patients to the internet or other sources of information (yes/effective 48%; no/not effective 6%; don’t know 46%).

**Table 3:** Percent Distribution of Nurse Practitioners’ Opinions about the Effectiveness of Recommended Communication Techniques.

Domain & Item	N	Yes (effective) (%)	No (not effective) (%)	Do not know (%)
***Interpersonal communication***				
**Limit number of concepts presented at a time to 2–3**^a^	161	78.26	1.86	19.25
**Ask patients whether they would like a family member or friend to accompany them in the discussion**^a^	151	35.10	2.65	62.25
**Draw pictures or use printed illustrations**^a^	158	74.68	1.90	23.42
**Speak slowly**^a^	161	82.61	1.86	15.53
**Use simple language**^a^	160	88.13	0.63	11.25
***Teach-back***				
**Ask patients to repeat back information or instructions**^a^	161	80.12	1.86	18.01
**Ask patients to tell you what they will do at home to follow instructions**^a^	161	65.84	6.83	27.33
***Patient-friendly materials and aids***				
**Use video or DVD**	145	28.28	5.52	66.21
**Hand out printed materials**	159	27.67	4.40	67.92
**Use models or x-rays to explain**	154	61.69	3.25	35.06
***Assistance***				
**Underline key points on print materials**	154	36.36	1.30	62.34
**Follow-up with patients by telephone to check understand and adherence**	151	31.13	2.65	66.23
**Read instructions out loud**	157	66.88	8.92	24.20
**Ask other office staff to follow-up with patients for post-care instructions**	151	53.64	5.30	41.06
**Write or print out instructions**	158	80.38	1.27	18.35
***Patient-friendly practice***				
**Refer patients to the internet or other sources of information**	156	48.08	5.77	46.15
**Use a translator or interpreter when needed**	154	78.57	0	21.43

^a^Seven basic communication techniques.

### Analysis of results by provider and practice characteristics

The following characteristics were not significantly associated with differences in mean numbers of communication techniques used, out of the 7 basic communication techniques and out of the 17 techniques, according to the bivariate analysis: year of graduation, race/ethnicity, sex, ever taken a communication course in addition to nursing school, inclusion of children in practice versus no children in practice, percentage of child patients with Medicaid, and practice setting (Table 4). The only significant relationship in the bivariate analysis of variance is between the NPs who did and did not report assessing the office for user-friendliness on predicting the mean number of communication techniques routinely used out of the seven basic communication techniques (4.60 techniques vs. 4.00 techniques, p < .01). That is, the repondents who reported assessing the office for user friendliness reported using a higher mean number of the seven basic communication techniques compared to those who reported they did not assess the office for user friendliness.

**Table 4:** Bivariate Analysis of Variance on Predictor Variables and Mean Number of Communication Techniques Routinely Used by Nurse Practitioners.

Variable	Sample Size	17 Communication Techniques		7 Basic Communication Techniques	
		Mean No of Techniques Used	Analysis of Variance (P-value)	Mean No of Techniques Used	Analysis of Variance (P-value)
**Provider Characteristics**					
***Year of graduation***	191		.09		.13
**1961–1977**	49	9.40		5.10	
**1978–1987**	51	9.00		4.40	
**1988–1997**	40	7.80		4.23	
**1998–2009**	51	8.22		4.00	
***Race/ethnicity***	191		.10		.22
**White**	161	9.84		4.22	
**Black**	19	9.84		5.00	
**All other**	11	9.00		4.40	
***Gender***	193		.10		.07
**Female**	181	9.00		4.40	
**Male**	12	7.00		3.50	
***Ever taken a communication course***	194		.70		.82
**No**	66	8.33		4.30	
**Yes**	128	9.10		4.34	
***Assessed office for user-friendliness***	192		.07		<.01
**No**	87	8.00		4.00*	
**Yes**	105	9.00		4.60*	
**Practice Characteristics**					
***Percentage of child patients with Medicaid***	127		.70		.60
**0%-25%**	56	8.00		4.11	
**26%-50%**	25	8.04		4.00	
**51%-75%**	16	8.30		4.30	
**76%-100%**	30	9.00		4.33	
***Practice setting***	193		.53		.96
**Solo practice**	17	9.20		5.0	
**Group practice**	69	8.11		4.30	
**Hospital**	32	9.10		4.30	
**Public health practice**	36	8.22		4.30	
**All others**	39	8.44		4.26	

*p <.01.

The ordinary least squares regression results of provider and practice characteristic predictor variables on the number of communication techniques used per week by nurse practitioners confirmed the findings of the bivariate analysis for all characteristics except assessing the practice for user-friendliness and having taken a communication course (Table 5). Non-significant predictors included: year of graduation, race/ethnicity, sex, group practice/hospital/public health settings versus solo practice, and percentage of pediatric Medicaid patients in practice. Assessing the office for user-friendliness did not demonstrate a significant difference in the average number of communication techniques used per week out of the 7 basic or the 17 total communication techniques in the ordinary least squares regression (p = .80), yet did demonstrate significance in the bivariate analysis on the mean number of techniques used out of the seven basic communication techniques (4.00 vs. 4.60 techniques, p < .01) (Table 4). Nurse practitioners who took a communication course in addition to nursing school used a higher number of the 7 basic communication techniques per week than the NPs who had not taken a communication course (Coefficient .97, SE .31, p < .01) (Table 5) in the ordinary least squares regression model, but this significance was not demonstrated in bivariate analysis (p = .82) (Table 4).

**Table 5:** Ordinary Least Squares Regression Results of Predictor Variables on Number of Recommended Communication Techniques Routinely Used by Nurse Practitioners.

Variable	17 Communication Techniques (N = 194)		7 Basic Communication Techniques (N = 194)	
	Coefficient (Standard Error)	P-value	Coefficient (Standard Error)	P-value
**Provider Characteristics**				
***Year of graduation***				
**1961–1977**	.80 (.90)	.38	.43 (.44)	.34
**1978–1987**	.42 (.86)	.63	.40 (.42)	.35
**1988–1997**	-.53 (.93)	.57	.30 (.50)	.50
**1998–2009**	Ref		Ref	
***Race/ethnicity***				
**Black vs. White**	2.10 (1.11)	.06	.80 (.54)	.14
**Other vs. White**	-0.70 (1.50)	.70	-.90 (.71)	.23
**White**	Ref		Ref	
**Female(Ref) vs. male**	-0.61 (1.30)	.64	.06 (.63)	.93
***Ever taken a communication course***				
**No(Ref) vs. yes**	1.20 (.63)	.07	.97 (.31)*	< .01
***Assessed office for user-friendliness***				
**No(Ref) vs. yes**	0.33 (.70)	.63	-.10 (.32)	.80
***Practice Characteristics***				
***Practice setting***				
**Other vs. group**	-0.14 (0.90)	0.87	-0.60 (.44)	.20
**Solo vs. group**	1.62 (1.10)	.13	0.63 (.52)	.23
**Hospital vs. group**	0.60 (0.95)	.53	-0.22 (.50)	.64
**Public Health vs. group**	-0.70 (1.00)	.50	-0.40 (.50)	.41
**Group**	Ref		Ref	
***Percentage of child patients with Medicaid***				
**0–25%**	-.02 (.90)	.98	-.07 (.44)	.90
**26–50%**	.35 (.95)	.71	.00 (.46)	.99
**51–75%**	.13 (1.02)	.90	.03 (.49)	.95
**76–100%**	Ref		Ref	

*p < .01.

## Discussion

### Routine use of communication techniques

NPs report that they are communicating in expected ways most of the time as demonstrated by the reported frequent use of simple language, use of teach-back method, and use of an interpreter when necessary. The quality of self-reported ‘simple language’ could be explored more and warrants further investigation, such as observation of actual patient encounters by video or audio-recording with qualitative analysis may be lower than the self-reported frequency [26]. In contrast to 30% of Maryland family physicians and pediatricians, 54% of NPs reported routinely using the teach-back method with patients [2].

### Opinions about effectiveness of communication techniques

Opinions about the effectiveness of communication techniques corresponds to the concept of outcome expectancies, a psychological determinant of behavior, which when higher lead to higher likelinhood to perform a behavior in social cognitive theory [42]. Thus, the higher proportions of respondents who believe in the effectiveness of limiting to 2 or 3 concepts presented, speaking slowly, and using simple language correspond theoretically to outcome expectancies of using those communication techniques more routinely. Furthermore, four of the 17 communication techniques also had high proportions of respondents state that they did “not know” about the effectiveness of the techniques, and these outcome expectancies theoretically correspond to lower routine use of these communication techniques. Evidence regarding the effectiveness of using a video or DVD, handing out printed materials, underlining key points on print materials, and following-up with patients by telephone to check understanding and adherence needs to be further established and disseminated in the literature. An explanation for the high proportion of respondents who stated they did “not know” about the effectiveness of asking a family member or friend to accompany them in the discussion may be be due to wording of the item being unrelated to actual clinical practice, wherein those respondents who routinely care for infants and children usually experience an adult caregiver accompanying a child patient and therefore they do not ask a child patient about desires to be accompanied. Further analytical studies could examine statistical correlations of theoretical relationships between opinions about the effectiveness of communication techniques and their routine use.

### Factors affecting use of communication techniques

Two statistical models of NP provider and practice characteristics demonstrated no significant difference for all sociodemographic characteristics. Potential significant predictors for differences in either mean number or in total number of communication techniques routinely used by nurse practitioners were: assessing the office for user-friendliness and taking a communication course. Because these variables were significant in one of the two models each, these findings could be considered preliminary, yet consistent with findings of reported communication techniques by other healthcare professionals in Maryland. These NP respondent had a similar rate (65%) of reporting having ever taken a communication course in addition to their nursing education compared to reported rates of dental hygienists (66%), dentists (60%) and physicians (50%) in Maryland [2,4,6]. Physicians in Maryland who had taken a communication course in addition to their education, compared to those who had not, reported using more communication techniques routinely [2]. Both variables of assessing the office for user-friendliness and taking a communication course in addition to their education predicted reported use of greater numbers of communication techniques routinely used according to surveys of dentists and dental hygienists in Maryland [4,6]. Assessing a healthcare facility for user-friendliness is an important component of health-literate organizations [3,33,39].

### Strengths

This study adds self-reported descriptions of NPs’ communication techniques to the Maryland state-wide oral health literacy assessment of dentists, dental hygienists, physicians, and nurse practitioners. The Maryland NP survey respondents routinely use several patient-friendly interpersonal communication techniques. The number of NPs who voluntarily responded “don’t know” when asked about the effectiveness of particular communication techniques speaks to high probability of honest reporting in those items, as well as high potential for honest reporting in other survey items.

The results provide direction for further education of NPs and other healthcare providers in areas for improvement, such as further enhancement of the skills in the domains of the use of patient-friendly materials and aids, patient assistance, and patient-friendly practices. Additional research regarding the effectiveness of these techniques would also enhance our current understanding because the providers’ opinions regarding effectiveness, conceptualized as outcome expectancies, is theoretically associated with the frequency or consistency with which a communication technique is used [43]. This work is aligned with Healthy People 2020 objectives associated with patient-centered and health-literate communication “to increase the proportion of persons who report that their healthcare providers have satisfactory communication skills” [25].

### Limitations

This was a self-reported survey, which may include some inherent potential for bias to report communication behaviors as performed more frequently than actually performed. Anonymity and confidentiality of responses with a mail-in survey method were used to decrease the potential for social desirability bias.

The draft of the survey questionnaire was reviewed by 6 clinicians with nursing backgrounds, but they were not all NPs. Some items related to educational background of NPs could have better reflected the complexity of today’s options for nurses to initially enter into practice, and then achieve higher degrees or certifications. Psychometric testing of the survey as conducted by the AMA and the ADA has not been done to our knowledge. Future studies could perform psychometric testing on domains with sufficient numbers of items.

The homogeneity of nurse practitioners may be similar to that of physicians, and thus nonresponse bias may be only a small threat [44]. The response rate of 21% was fairly low, although consistently similar to the recent low response rates of physicians and dentists in Maryland [2,6]. Over the past 20 years, low response rates have been typical of healthcare professionals’ surveys, even with incorporation of recommended strategies to boost response rates like we used, such as multiple mailings and endorsement by local professional leaders [44–46]. This sample is very slightly over-represented by NPs in Maryland who provide services to children with Medicaid as the primary pediatric dental insurance (43% of sample total) compared to a state-wide average of 32.6% of Maryland’s children who are enrolled in Medicaid [47]. Two statistical models, rather than one model, and cautious interpretation were used to control for potential selection bias.

## Conclusions

This survey of self-reported use of communication techniques of nurse practitioners contributes greater understanding to a state-wide oral health literacy assessment of several healthcare professionals in Maryland. Nearly all NPs (95.9%) reported using simple language. More than 80% of NPs routinely used speaking slowly and limiting content to 2 or 3 main points. Approximately half of the NPs reported routine use of the teach-back method. Similar to survey findings of other health professional in Maryland, our findings suggest that nurse practitioners who had assessed the healthcare office for patient-friendliness or who had taken a communication course beyond their initial education are positive predictors for using more of the 7 basic communication techniques. These self-reported findings should be validated with observational research using qualitative analysis of NP-patient encounter transcripts.

Graduate education and continuing education for NPs should increase and continue emphasis on health-literate and patient-centered communication techniques. Emphasis could include assessing the environment of the practice for patient-friendliness to develop more health-literate organizations and communication techniques recommended by AHRQ’s *Health Literacy Universal Precautions Toolkit* [33,38]. This study provides a baseline assessment of self-reported NP communication practices which are expected evolve along with national healthcare reform for improved patient safety and quality of care.

## Supporting Information

S1 Data Open-access data availability. Data is available as a Supporting Information File.
(SAV)

## References

[1] Smith W, Brach C, Horowitz AM. Poor oral health literacy: why nobody understands you. J Dent Hyg. 2015;89 Suppl 1:36–38. 25691025 PMC5102013

[2] Weatherspoon DJ, Horowitz AM, Kleinman DV, Wang MQ. Maryland physicians’ use of recommended communication techniques. PLoS ONE. 2015;10(4): e0119855. 25856371 10.1371/journal.pone.0119855 PMC4391842

[3] Horowitz AM, Maybury C, Kleinman DV, Radice SD, Wang MQ, Child W, et al Health literacy environmental scans of community-based dental clinics in Maryland. Am J Public Health. 2014;104(8): e85–93. 10.2105/AJPH.2014.302036 24922128 PMC4103217

[4] Horowitz AM, Clovis JC, Wang MQ, Kleinman DV. Use of recommended communication techniques by Maryland dental hygienists. J Dent Hyg. 2013;87(4): 212–23. 23986414

[5] Horowitz AM, Kleinman DV, Wang MQ. What Maryland adults with young children know and do about preventing dental caries. Am J Public Health. 2013;103:e69–e76. 10.2105/AJPH.2012.301038 PMC3698752 23597372

[6] Maybury C, Horowitz AM, Wang MQ, Kleinman DV. Communication techniques used by Maryland dentists. J Am Dent Assoc. 2013;144(2): 1386–96. 10.14219/jada.archive.2013.0075 24282269

[7] Clovis JB, Horowitz AM, Kleinman DV, Wang MQ, Massey M. Maryland dental hygieeinists' knowledge, opinions and practices regarding dental caries prevention and early detection. J Dent Hyg. 2012;86:292–305. 23168104

[8] U.S. Department of Health and Human Services, National Institutes of Health, National Institute of Dental and Craniofacial Research. The invisible barrier: literacy and its relationship with oral health. J Public Health Dent. 2005;65(3): 174–82. 16171263 10.1111/j.1752-7325.2005.tb02808.x

[9] Brach C, Dreyer B, Schyve P, Hernandez LM, Baur C, Lemerise AJ, et al. Attributes of a health literate organization. 2012 Jan 27. Available: http://nam.edu/perspectives-2012-attributes-of-a-health-literate-organization/. Accessed 4 August 2015.

[10] Otto M. For want of a dentist. Washington Post. 28 Feb 2007. Available: http://www.washingtonpost.com/wp-dyn/content/article/2007/02/27/AR2007022702116.html. Accessed 4 August 2015.

[11] Institute of Medicine. Advancing oral health in America. Washington: National Academies Press; 2011.

[12] Satcher DS. Surgeon General's report on oral health. Public Health Reports. 2000;115(5): 489–90. 11236021 10.1093/phr/115.5.489 PMC1308610

[13] Gift HC, Reisine ST, Larach DC. The social impact of dental problems and visits. Am J Public Health. 1992;82(12): 1663–8. 1456343 10.2105/ajph.82.12.1663 PMC1694558

[14] Sanders B. U.S. Senate Committee on Health, Education, Labor, & Pensions, Subcommittee on Primary Health and Aging. Dental crisis in America: the need to expand access 29 Feb 2012. Available: http://www.sanders.senate.gov/imo/media/doc/DENTALCRISIS.REPORT.pdf. Accessed 4 August 2015.

[15] American Academy of Pediatric Dentistry Council on Clinical Affairs. Policy on the dental home. Pediatr Dent. 2008;30(7 Suppl): 22 19216373

[16] Hale KJ. Oral health risk assessment timing and establishment of the dental home. Pediatrics. 2003;111(5): 1113–6. AAP Policy Statement reaffirmed May 2009; 124. 12728101 10.1542/peds.111.5.1113

[17] American Academy of Nurse Practitioners. Scope of Practice for Nurse Practitioners. AANP. 2013. Available: http://www.aanp.org/images/documents/publications/scopeofpractice.pdf. Accessed 4 August 2015.

[18] U.S. Congress. Patient Protection and Affordable Care Act, Public Law No. 111–148, 2702, 124 Stat. 119. 2010.

[19] Koh HK, Berwick DM, Clancy CM, Baur C, Brach C, Harris LM, et al New federal policy initiatives to boost health literacy can help the nation move beyond the cycle of costly 'crisis care' Health Aff (Millwood). 2012;31:434–443. 22262723 10.1377/hlthaff.2011.1169 PMC5102007

[20] Millenson ML, Macri J, eds. *Will the Affordable Care Act Move Patient-Centeredness to Center Stage*? http://www.rwjf.org/content/dam/farm/reports/reports/2012/rwjf72412 ed. Robert Wood Johnson Foundation and the Urban Institute; 2012.

[21] Weinstein RS, Graham AR, Erps KA, Lopez AM. Health literacy: the Affordable Care Act ups the ante. *Am J Med*. 2013;126:1029–1030. 10.1016/j.amjmed.2013.06.030 24262720

[22] The Joint Commission. *Advancing effective communication*, *cultural competence*, *and patient- and family-centered care*: *A roadmap for hospitals*. Oakbrook Terrace, IL: The Joint Commission; 2010.

[23] National Quality Forum. *Safe practices for better healthcare—2010 update*. 2010.

[24] Berkman ND, Sheridan SL, Donahue KE, Halpern DJ, Viera A, Crotty K, et al. Health Literacy Interventions and Outcomes: An Updated Systematic Review. Evidence Report/Technology Assesment No. 199. (Prepared by RTI International–University of North Carolina Evidence-based Practice Center under contract No. 290-2007-10056-I. AHRQ Publication Number 11-E006. Rockville, MD. Agency for Healthcare Research and Quality. March 2011.

[25] U.S. Department of Health and Human Services [HHS]. Office of Disease Prevention and Health Promotion. (2010). Health communication and health information technology objectives. Healthy People 2020

[26] Berry JA. Nurse practitioner/patient communication styles in clinical practice. J Nurse Pract. 2009;5(7): 508–15. 10.1016/j.nurpra.2009.02.019

[27] Hart V. Communication and Nursing: historical roots and related theory In: Hart V, editor. Patient-Provider Communications: Caring to Listen. Sudbury: Jones & Bartlett; 2010 pp 1–41.

[28] Hudon C, Fortin M, Haggerty JL, Lambert M, Poitras M. Measuring Patients’ Perceptions of Patient-Centered Care: A Systematic Review of Tools for Family Medicine. *The Annals of Family Medicine*. 2011;9:155–164. 10.1370/afm.1226 21403143 PMC3056864

[29] Charlton CR, Dearing KS, Berry JA, Johnson MJ. Nurse practitioners' communication styles and their impact on patient outcomes: an integrated literature review. J Am Acad Nurse Pract. 2008;20(7): 382–8. 10.1111/j.1745-7599.2008.00336.x 18638178

[30] Gilbert DA, Hayes E. Communication and outcomes of visits between older patients and nurse practitioners. Nurs Res. 2009;58(4): 283–93. 10.1097/NNR.0b013e3181ac1413 19609180 PMC2882707

[31] Paterniti D, Fancher T, Cipri C, Timmermans S, Heritage J, Kravitz R. Strategies primary care physicians use to deny patient requests. Arch Intern Med. 2010;170(4): 381–8. 10.1001/archinternmed.2009.533 20177043 PMC4090224

[32] Schwartzberg JG, Cowett A, VanGeest J, Wolf MS. Communication techniques for patients with low health literacy: a survey of physicians, nurses, and pharmacists. Am J Health Behav. 2007;31(1 Supple): S96–104. 17931143 10.5555/ajhb.2007.31.supp.S96

[33] Brega AG, Barnard J, Mabachi NM, Weiss BD, DeWalt DA, Brach C, et al. AHRQ Health Literacy Universal Precautions Toolkit (2nd ed). AHRQ Publication No. 15-0023-EF. Rockville: Agency for Healthcare Research and Quality. 2015 Available: http://www.ahrq.gov/professionals/quality-patient-safety/quality-resources/tools/literacy-toolkit/healthlittoolkit2.pdf. Accessed 2 July 2015.

[34] Plain Language Action and Information Network (PLAIN). What is plain language? In PlainLanguage.gov: Improving communication from the federal government to the public, 2004. Available: http://www.plainlanguage.gov/whatisPL/index.cfm. Accessed 1 December 2015.

[35] White M, Garbez R, Carroll M, Brinker E, Howie-Esquivel J. Is 'teach-back' associated with knowledge retention and hospital readmission in hospitalized heart failure patients? *J Cardiovasc Nurs*. 2013;28(2):137–146. 10.1097/JCN.0b013e31824987bd 22580624

[36] Wilson FL, Baker LM, Nordstrom CK, Legwand C. Using the teach-back and orem's self-care deficit nursing theory to increase childhood immunization communication among low-income mothers. *Issues Compr Pediatr Nurs*. 2008;31(1):7–22. 10.1080/01460860701877142 18300059

[37] Negarandeh R, Mahmoodi H, Noktehdan H, Heshmat R, Shakibazadeh E. Teach back and pictorial image educational strategies on knowledge about diabetes and medication/dietary adherence among low health literate patients with type 2 diabetes. *Primary Care Diabetes*. 2013;7(2):111–118. 10.1016/j.pcd.2012.11.001 23195913

[38] Schillinger D, Piette J, Grumbach K, Wang F, Wilson C, Daher C, et al Closing the loop: physician communication with diabetic patients who have low health literacy. Arch Intern Med. 2003;163(1): 83–90. 10.1001/archinte.163.1.83 12523921

[39] Rudd R, Anderson J. The health literacy environment of hospitals and health centers Partners for action: making your healthcare facility literacy-friendly. Boston: Harvard School of Public Health; 2006 Available: http://cdn1.sph.harvard.edu/wp-content/uploads/sites/135/2012/09/healthliteracyenvironment.pdf. Accessed 4 August 2015.

[40] Forrest JL, Horowitz AM, Shmuely Y. Caries preventive knowledge and practices among dental hygienists. J Dent Hyg. 2000;74(3): 183–95. 11318004

[41] Loupe MJ, Frazier PJ, Horowitz AM, Kleinman DV, Caranicas PC, Caranicas DA. Impact of an NIDR educational program on the teaching of caries prevention in dental hygiene. J Dent Educ. 1988;52(3): 149–55. 3422653

[42] Rozier RG, Horowitz AM, Podschun G. Dentist-patient communication techniques used in the United States: the results of a national survey. J Am Dent Assoc. 2011;142(5): 518–30. 10.14219/jada.archive.2011.0222 21531934

[43] Williams DM. Outcome Expectancy and Self-Efficacy: Theoretical Implications of an Unresolved Contradiction Personality and Social Psychology Review. 2010;14:417–425. 10.1177/1088868310368802 20505161

[44] Kellerman SE, Herold J. Physician response to surveys: a review of the literature. Am J Prev Med. 2001;20(1): 61–67. 10.1016/S0749-3797(00)00258-0 11137777

[45] Cook JV, Dickinson HO, Eccles MP. Response rates in postal surveys of health professionals between 1996 and 2005: an observational study. BMC Health Serv Res. 2009;9: 160 10.1186/1472-6963-9-160 19751504 PMC2758861

[46] VanGeest JB, Johnson TP, Welch VL. Methodologies for improving response rates in surveys of physicians: a systematic review. Eval Health Prof. 2007;30(4): 303–321. 10.1177/0163278707307899 17986667

[47] American Academy of Pediatrics. Maryland Medicaid facts. American Academy of Pediatrics. 2015. Available: https://www.aap.org/en-us/Documents/federaladvocacy_medicaidfactsheet_maryland.pdf. Accessed 2 July 2015.

